# The acute hypoalgesic effects of active head-mounted display virtual reality games

**DOI:** 10.1371/journal.pone.0308064

**Published:** 2024-08-14

**Authors:** Keith E. Naugle, Xzaliya A. Cervantes, Carolyn L. Boone, Brandon Wind, Kelly M. Naugle

**Affiliations:** 1 Department of Kinesiology, School of Health and Human Sciences, Indiana University Indianapolis, Indianapolis, Indiana, United States of America; 2 College of Osteopathic Medicine, University of Pikeville, Pikeville, Kentucky, United States of America; Università degli Studi di Milano: Universita degli Studi di Milano, ITALY

## Abstract

The purpose of this study was to determine: (1) whether physically active virtual reality (VR) games exert an acute hypoaglesic effect on the thigh and bicep compared to a non-active VR game and an exercise only condition matched for exercise intensity in healthy individuals, and (2) whether movement variables during gameplay are associated with the hypoalgesic effect of the games. Twenty young adults completed five separate study sessions, with each session devoted to playing one head-mounted display VR game or stationary cycling for 15 minutes. The games included Holopoint at level 2 and level 3, Hot Squat, and Relax Walk. Pressure pain thresholds at the thigh and bicep were measured pre and post VR gameplay and cycling. Participants wore a heart rate monitor and accelerometers on the wrist and thigh during play to measure the intensity and quantity of movement. Repeated measures ANOVAs revealed that pressure pain thresholds on the bicep increased from pre to posttest for each condition. The results also revealed that pressure pain thresholds on the thigh increased only for the conditions eliciting the greatest cardiovascular response, which included Holopoint at level 3, Hot Squat, and cycling. Bivariate correlations indicated that moderate to vigorous physical activity of the thigh was associated with pain reduction at the thigh during Holopoint. These results revealed that active VR games and exercise exerted a more widespread hypoalgesic effect compared to the non-active VR game, which was likely driven in part by the intensity and quantity of movement during gameplay.

## Introduction

Accumulating research suggests that virtual reality (VR) exerts a hypoalgesic effect via a distraction mechanism in healthy and clinical populations [1–4]. For example, recent studies show that passively engaging in the VR environment via a head-mounted display acutely reduces pain sensitivity and perception of experimentally induced pain in healthy adults [2, 4, 5]. Immersion in the VR environment likely provides a position distraction from painful stimuli, thereby attenuating pain. With new advances in VR technology, commercial game developers have increasingly released VR games which allow participants to interact with the VR environment via physical movements (active VR games). Recent research suggests that several commercial VR active games, played through a head-mounted display and controllers, induce a moderate intensity level of physical activity [6, 7] providing the opportunity to use these games as a form of exercise. Less is known about the hypoalgesic effects of physically active vs. non-active VR.

A significant body of evidence suggests that an acute bout of moderate to vigorous aerobic or isometric exercise induces a hypoalgesic effect to experimentally induced pain in healthy individuals [8]. Exercise induced hypoalgesia (EIH) can be generated by central (pain reduction in non-exercising body part) and local (pain reduction in exercising body part) pain inhibitory effects with the underlying mechanisms likely involving modulation of the opioid, serotonergic, and endocannabinoid systems [9, 10]. Given the separate hypoalgesic effects of exercise and VR, physically active VR games could potentially reduce pain via multiple mechanisms. To our knowledge, only one study has evaluated the acute hypoalgesic effects of physically active vs. passive VR games on experimentally induced pain [11]. This study found that the active and non-active VR games reduced pain sensitivity on the forearm; however, the VR game eliciting the most whole-body movement exerted the greatest hypoalgesic effect on the leg. This study did not include a physical activity only condition. Therefore, it remains unclear whether VR combined with physical activity exerts a pain reducing effect above and beyond physical activity (without VR) performed at a similar intensity. Additionally, no research has evaluated the degree to which movement of the active VR games contributes to their hypoalgesic effect.

The purpose of this study was to determine whether physically active VR games, played via a head-mounted display, exert an acute hypoaglesic effect on the thigh and bicep compared to a non-active VR game and an exercise only condition (matched for exercise intensity to the active VR) in healthy individuals. We hypothesized that the active VR games would have a greater hypoalgesic effect than non-active VR and exercise without VR. Secondly, we examined whether the quantity and intensity of movement during the active VR games was associated with the hypoalgesic effect during active VR gameplay. We hypothesized that greater time in MVPA during gameplay would be associated with greater reduction in pain sensitivity.

## Methods

### Participants

Twenty-one participants between the ages of 18 and 34 enrolled in this study. The recruitment period for participants took place from September 20, 2021, to January 30 2023. A power analysis using G Power 3.1.9.7 was used to estimate the sample size needed for detecting a within subject difference in pressure pain threshold (PPT) scores between conditions and PPT tests. With power set at 0.80, alpha at 0.05, a 0.5 correlation among repeated measures, and an estimated moderate effect size of *f* = .25, the power analyses indicated that a minimum of 15 participants was needed. All participants completed a written IRB-approved informed consent form prior to study participation. Participants were recruited from the local university and community with posted study advertisements. Inclusion criteria was ages 18–35 years old. Exclusion criteria included proneness to motion sickness or claustrophobia, the presence of an acute or chronic pain condition, and an answer of “yes” on any of the general health questions on the Physical Activity Readiness Questionnaire (PAR-Q+ 2019 version) [12]. Participants were also asked to fast 1 hour before each session, to not consume alcohol for 24 hours prior to sessions, and to refrain from vigorous exercise and ingesting caffeine and analgesic medications on the day of the sessions prior to the session.

#### Procedures

This study used a repeated measures design in which participants completed all conditions. Participants completed five study visits on separate days. This study was approved by the Indiana University Institutional Review Board (IRB).

*Enrollment*, *Screening*, *and Familiarization (Visit 1)*. The first study visit included the informed consent process, screening with the completion of the PAR-Q+ and demographics questionnaire, and completion of the International Physical Activity Questionnaire-Short Form (IPAQ-SF). The demographics questionnaire assessed VR playing experience with the following scale: 0 = Never, 1 = Equal to or less than one time per week, 2 = two to four times per week, and 3 = five or more times per week. After study eligibility was confirmed, participants sat quietly for 10 minutes to collect resting heart rate (HR). Participants were then familiarized with and practiced the pressure pain threshold (PPT) test used to measure pain sensitivity. The experimenter explained the PPT test procedures and then three practice trials were performed on each of the participants’ non-dominant forearm and thigh. Then, participants were shown the Meta Quest 2 VR system (Menlo Park, CA) which includes a headset and two handheld controllers. Each participant was individually fitted with the headset. The VR headset is designed to track the movement of the head and controllers and then translate those movements into the 3-dimensional VR environment. During VR familiarization (~5–10 minutes), participants were shown the different buttons on the controllers and then wore the headset to get acclimated to having the headset on. Once the headset was on, participants were verbally led by the experimenter on how to use the controllers to navigate the VR environment. Participants were also shown how they would be made aware of the boundaries of the play space while wearing the headset (i.e., virtual walls appear when the participant gets too close to the edge of the play area).

*Visits 1–4*. Participants played one VR game during each study visit, which included Holopoint, Hot Squat, and Relax Walk. Holopoint was played in two separate visits, but at different levels. One visit was played at level 2 (L2) and one visit was played at level 3 (L3). We have previously shown that Holopoint played at L3 elicits greater cardiovascular intensity compared to L2 [13]; therefore, we expect different levels of movement to be achieved in L2 vs. L3 of this game. This variation in movement during gameplay is important for testing our second hypothesis that the quantity and intensity of movement during the active VR games would be associated with the hypoalgesic effect. See Table 1 for description of the games. Relax Walk is a non-active VR game, while Holopoint and Hot Squat require significant movement. Participants played the games at a self-selected intensity. All games were played in a 6.5 x 8.5 feet space. The order of games was randomized.

**Table 1:** Description of VR games.

VR Game	Description
Holopoint (Active)	Holopoint is an archery game that requires participants to use the controllers as a bow and arrow to hit objects that are initially stationary but eventually fly at the participant. Once the targets are hit by an arrow, participants have to dodge the projectiles that launch back at the participant. The speed and volume of targets increase from level 2 to level 3.
Hot Squat (Active)	Hot Squat is a game to music in which participants have to repeatedly perform squats, and at times sustain a squat, to avoid hitting the ceiling of incoming tunnels.
Relax Walk (non-active)	Relax Walk is a nonactive game in which participants explore virtual nature environments using the controller to move from place to place.

**Fig 1:**
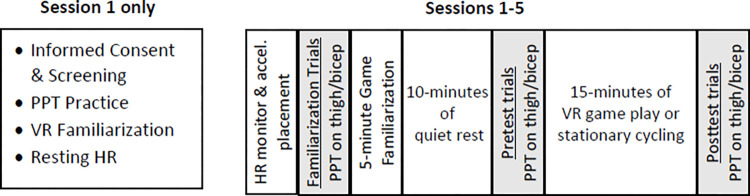
Order of experimental events. Testing of the pressure pain sensitivity is marked by the lightly shaded boxes. Accel = accelerometer, PPT = pressure pain threshold.

See Fig 1 for the order of experimental events during each session. Prior to game play, participants were fitted with a Polar HR monitor and accelerometers worn on the dominant wrist and ipsilateral thigh. Then, participants played the game for 5 minutes for familiarization and then sat quietly for 10 minutes to allow HR to return to resting. Next, participants played the game for 15 minutes. PPT’s were administered three separate times during each session as shown in Fig 1: 1) familiarization trials—before game familiarization (without headset on), 2) Pretest—after 10 minutes of rest, just prior to the 15-minute gameplay (without headset on), and 3) Posttest—immediately after the 15-minute gameplay while the headset was still on. The familiarization trials were conducted to 1) refamiliarize the participants with the PPT test, and 2) to allow examination of repeated pain testing effects.

*Visit 5 –Physical activity session*. The physical activity session had an identical protocol to visits 2–4, except the 15-minute period of VR was replaced by 15 minutes of riding a stationary bicycle (Cycle Ops Power, Saris, Madison WI) at a predetermined intensity. During the 5 minutes of familiarization, participants rode the bike at a very light intensity. During the 15-minute period, participants were asked to ride the bike at a similar intensity (based on HR) as the highest intensity played during VR game play. For example, if a participant played Hot Squat at the highest aerobic intensity based on HR, then the average HR during Hot Squat for minutes 1–5, 6–10, and 11–15 were determined for that participant. If the average HR for Hot Squat for minutes 1–5 was 126 beats, then the participant was given a target HR range of 121–131 (average +/-5) for minutes 1–5 on the bike. Participants were instructed to bike faster or slower to keep their HR within the target range.

### Outcome measures

#### Pressure pain threshold (PPT)

PPT’s of the thigh and bicep were assessed with a pressure algometer (Wagner Instruments, Greenwich, CT) with a 1 cm rubber tip placed on the skin’s surface. The experimenter gradually applied pressure to the skin until the participant signaled the first sensation of pain, at which time the algometer was removed. PPT was defined as the amount of pressure in foot-pounds at which the participant reported the first sensation of pain. Two consecutive trials were performed at each body site at each time point (familiarization trials, pretest, posttest) while the participant was in a seated position with the knees at a 90° angle and the dominant arm lying flat on an adjacent table. The specific body sites included 10 cm above the knee on the dominant thigh and the midpoint on the dominant bicep between the axilla and elbow crease. Intertrial intervals were 20 seconds. The order of the PPT’s at each body site was counterbalanced across participants. However, each participant retained the same body site order across games. The average PPT value from the two trials for each time point and body site was used in statistical analyses. Also, a change score was calculated to assess the magnitude of change in PPTs from pretest to posttest (PPT posttest–PPT pretest).

#### Minutes in cardiovascular MVPA (MVPA-HR)

Heart rate was measured every second during gameplay with a Polar HR monitor (Polar, Kempele, Finland). Heart rate values were used to calculate the percentage of HR reserve (%HRR) achieved each second of game play with the following formula: [(HR during activity–resting HR)/HRR] x 100 [14]. Percentage of HRR values that are ≥ 40% are considered at least moderate intensity [14–16]. We then calculated the number of minutes that participants reached ≥ 40% of HRR during game play (MVPA-HR). Additional HR data for the games is published in Naugle et al [13].

#### Percentage of sedentary time and MVPA of the arm and thigh

Participants wore ActiGraph GT3X+ (Pensacola FL) accelerometers on the dominant wrist and ipsilateral thigh during gameplay. Activity count data was captured in 1-second epochs and processed with the ActiLife software. The following activity count cut-points were used to determine the percentage of time participants spent in sedentary time and MVPA: sedentary < 100, MVPA >1951 [17]. When scoring the data, ActiGraph’s “worn on wrist” correction was applied for the wrist accelerometer data. The ActiGraph GT3X+ has shown to be valid and reliable in measuring physical activity [18, 19].

#### Statistical analyses

All data were analyzed using SPSS v. 29 (SPSS, Inc., Chicago, IL). Descriptive statistics were calculated for all the outcome variables. Preliminary analyses indicated that sex differences did not exist in the effect of the different conditions on the PPT’s; thus, we did not include sex as a factor in the ANOVAs. To evaluate the first hypothesis, PPTs of the bicep and thigh were analyzed with separate 5(condition: Holopoint L2, Holopoint L3, Hot Squat, Relax Walk, cycling) x 3(time: familiarization trials, pretest, posttest) repeated measures ANOVAs. One-way repeated measures ANOVAs were conducted on MVPA-HR and the accelerometers variables to determine the amount and intensity of PA during gameplay. Post hoc analyses were conducted with simple effects tests for significant interactions, and t-tests with Bonferroni corrections for significant main and simple effects. If the sphericity assumption was violated, then Greenhouse-Geisser degrees of freedom corrections were applied to obtain the critical p-value. To evaluate the second hypothesis, Pearson’s bivariate correlations were conducted to determine the relationship between the accelerometer variables and MVPA-HR with the magnitude of the hypoalgesic responses for Hot Squat and Holopoint (L2 and L3 combined). The p-value for significance was set at p <0.05.

We also report partial eta squared (η_p_^2^), which measures an effect size in ANOVA models. The values for η_p_^2^ range from 0 to 1, with higher values indicating a greater proportion of variance that is associated with a given variable in the model after accounting for variance explained by other variables in the model. Partial eta squared values can be interpreted as 0.01 = small effect, 0.06 = medium effect, and ≥0.14 = large effect size [20].

## Results

### Descriptive characteristics

Twenty adults completed all study visits. Participant characteristics are presented in Table 2. One participant did not complete the exercise study session and therefore was excluded from data analysis. Scores from the IPAQ indicated that on average the study sample fell within the high physical activity category (>3000 MET*minutes/week). Overall, participants reported little (≤ 1 time per week) to no VR experience.

**Table 2:** Participant characteristics.

Sex, % female		40%
Race, %		
Caucasian		80%
Asian		5%
Hispanic		15%
Age, years		23.4 ± 4.5
IPAQ, total score		4655 ± 3676

*Note*. Data are presented as percentage or mean ± standard deviation.

### Pain sensitivity

#### PPT on the bicep

The ANOVA conducted on the bicep PPT’s showed a main effect of time, *p* = .004 (η_p_^2^ = 0.29). The follow-up comparisons indicated no changes from familiarization trials (*M* = 8.11±3.80) to the pretest (*M* = 8.15±4.05), but a significant increase from both the familiarization trials and the pretest to the posttest (*M* = 9.00±4.83). The main effect of condition (*p* = .108, η_p_^2^ = .10) and the time x condition interaction (*p* = .950; η_p_^2^ = 0.003) were not significant. See Table 3 for the PPTs by condition, time, and body site.

**Table 3:** Means and Standard Deviations (SD) for pressure pain thresholds across condition, time, and body site.

		Holopoint L2	Holopoint L3	Hot Squat	Relax Walk	Cycling
*Bicep*						
Pretest-1		7.4 ± 3.3	8.4 ± 5.3	8.3 ± 4.0	7.7 ± 3.5	8.8 ± 5.3
Pretest-2		7.6 ± 3.3	8.4 ± 4.6	8.2 ± 4.2	7.5 ± 3.7	9.0 ± 5.4
Posttest		8.5 ± 4.1	9.1 ± 5.2	9.2 ± 5.3	8.5 ± 5.4	9.8 ± 6.5
*Thigh*						
Pretest-1		16.8 ± 8.1	16.4 ± 7.9	15.6 ± 6.9	16.3 ± 6.7	17.3 ± 9.8
Pretest-2		15.4 ± 6.8	16.6 ± 8.9	16.7 ± 9.1	16.2 ± 7.9	17.3 ± 9.8
Posttest		17.0 ± 9.3	17.9 ± 9.7	18.1 ± 9.8	16.0 ± 7.8	20.6 ± 13.0

Note: L2 = Level 2, L3 = Level 3, Pressure pain threshold units lb*ft

#### PPT on the thigh

One participant did not report pain on the PPT test and thus was excluded from the analyses. The ANOVA conducted on the thigh PPTs showed a main effect of time (*p* = .014, η_p_^2^ = 0.26), which was superseded by a significant time x condition interaction, *p* = .019 (η_p_^2^ = 0.15). The simple effects of time were significant for Holopoint L3 (*p* = .029, η_p_^2^ = 0.34), Hot Squat (*p* = .022, η_p_^2^ = 0.36), and cycling (*p* = .008, η_p_^2^ = 0.43), but not Holopoint L2 (*p* = .07, η_p_^2^ = 0.26) and River Walk (*p* = .930, η_p_^2^ = 0.008). The significant follow-up comparisons indicated that PPT’s increased from pretest to posttest for Holopoint L3, Hot Squat, and cycling. PPTs also increased from the familiarization trials to posttest for cycling. No changes were evident from the familiarization trials to the pretest.

### Physical activity during gameplay

#### MVPA-HR

Heart rate did not collect correctly for two participants and thus their data was not included in the data analysis. The ANOVA showed a main effect of condition, *p* < .001 (η_p_^2^ = 0.73). Significant follow-up tests indicated the following differences in MVPA-HR: 1) Relax Walk elicited decreased MVPA-HR compared to all other conditions, 2) Hot Squat elicited greater MVPA-HR compared to Holopoint L2 and L3, and 3) Holopoint L3 and cycling elicited greater MVPA-HR compared to Holopoint L2. See Table 3 for physical activity variables. See Table 4 for the physical activity during gameplay data.

**Table 4:** Means and Standard Deviations (SD) for MVPA based on HR and accelerometer variables.

		Holopoint L2	Holopoint L3	Hot Squat	Relax Walk	Cycling
*MVPA based on HR*, *minutes*						
		4.3 ± 4.5	7.4 ± 5.0	11.8 ± 2.2	0.0 ± 0.0	11.1 ± 4.5
*Physical activity of dominant arm (wrist accelerometer)*, *% of time*						
Sedentary		3.3 ± 2.4	2.0 ± 2.3	21.4 ± 19.5	81.2 ± 18.7	74.3 ± 13.7
MVPA		87.5 ± 5.8	92.2 ± 6.6	53.8 ± 19.5	7.8 ± 9.7	9.4 ± 8.9
*Physical activity of dominant thigh (thigh accelerometer)*, *% of time*						
Sedentary		34.9 ± 19.9	30.2 ± 20.4	30.4 ± 15.6	93.6 ± 6.0	2.5 ± 6.9
MVPA		22.4 ± 19.5	25.9 ± 22.5	39.7 ± 20.1	0.4 ± 1.1	95.1 ± 9.1

Note: L2 = Level 2, L3 = Level 3

#### Percentage of sedentary time and MVPA of dominant arm

The analysis on sedentary time of the arm indicated an effect of condition, *p* < .001 (η_p_^2^ = 0.90). Follow-up tests revealed that 1) Relax Walk and cycling had significantly greater arm sedentary time compared to the 3 active VR games, and 2) Hot Squat had significantly greater arm sedentary time relative to Holopoint level 2 and 3. The ANOVA on MVPA of the arm was also significant (*p* < .001, η_p_^2^ = 0.94) with the following significant differences between conditions: Holopoint L3 > Holopoint L2 > Hot Squat > Relax Walk and cycling.

#### Percentage of sedentary time and MVPA of dominant thigh

The analysis on sedentary time of the thigh indicated an effect of condition, *p* < .001 (η_p_^2^ = 0.87). Follow-up tests revealed that Relax Walk had significantly greater thigh sedentary time compared to all other conditions and the 3 active games had greater thigh sedentary time compared to cycling. The ANOVA on MVPA of the thigh was also significant (*p* < .001, η_p_^2^ = 0.86), with the following significant differences between conditions: Relax Walk < 3 active games < cycling.

#### Relationships between magnitude of pain reduction and movement variables during active games

No significant correlations existed between the movement variables (i.e., accelerometer variables and MVPA-HR) and the magnitude of pain reduction at the arm and thigh for Hot Squat. For Holopoint (levels 2 and 3 combined), the magnitude of pain reduction at the thigh was significantly correlated with the thigh accelerometers variables of MVPA (*r* = .422, *p* = .008) and sedentary time (*r* = -.383, *p* = .018). Thus, greater MVPA and less sedentary time of the thigh during Holopoint was related to greater magnitude of pain reduction at the thigh. No significant correlations were found for the movement variables and the magnitude of pain reduction at the bicep (See Table 5).

**Table 5:** Bivariate correlations between magnitude of pain reduction and movement variables.

	Holopoint		Hot Squat	
Movement Variable	Bicep	Thigh	Bicep	Thigh
MVPA-HR	-0.027	0.233	-0.263	-0.182
Thigh MVPA, accelerometer	0.295	0.422**	-0.247	-0.042
Thigh sedentary time, accelerometer	-0.272	-0.383*	-0.064	0.304
Wrist MVPA, accelerometer	0.011	0.169	0.129	0.014
Wrist sedentary time, accelerometer	0.156	0.098	-0.225	-0.152

Note: HR = heart rate, MVPA = moderate to vigorous physical activity

**p < .01

*p < .05.

#### Summary of results

The repeated measures ANOVAs revealed that PPTs on the bicep increased from pre to posttest for each condition. The results also revealed that PPTs on the thigh increased from pre to posttest only for Holopoint at level 3, Hot Squat, and cycling. Hot Squat and cycling, followed by Holopoint L3, elicited the greatest cardiovascular MVPA. Bivariate correlations indicated that moderate to vigorous physical activity of the thigh was associated with pain reduction at the thigh during Holopoint.

## Discussion

This study was the first to evaluate whether physically active VR games have a similar hypoalgesic effect compared to both non-active VR and exercise performed at a similar intensity. Virtual reality reduces pain sensitivity through distraction mechanisms [1, 2], while an acute bout of exercise exerts local and central pain inhibitory effects through mechanisms likely involving activation of the endogenous opioid, serotonergic, and endocannabinoid systems [21, 22]. Active VR games provide the opportunity for participants to gain the beneficial physiological effects of physical activity while also providing a mode of distraction from unpleasant stimuli.

Our first hypothesis was that the active VR games would have a greater hypoalgesic effect compared to the non-active VR game and exercise without VR. This hypothesis was not supported by the PPTs on the bicep and partially supported by the PPTs on the thigh. As expected, we observed no change in PPTs following quiet rest (familiarization trials to pretest) for the thigh and bicep, suggesting no effect of repeated pain testing. However, pain sensitivity was reduced from the pretest to posttest following all conditions at the bicep, with no differences between conditions. Thus, non-active VR, active-VR, and exercise-alone exerted hypoaglesic effects at the bicep. This finding is similar to Evans et al. who showed decreases in pressure pain sensitivity at the forearm following active (i.e., Holopoint, Hot Squat, Beat Saber) and non-active VR (i.e., Relax Walk) [11].

Differing mechanisms could have accounted for the pain reduction at the bicep following each condition. For example, hypoalgesia following Relax Walk, which elicited very little movement, was likely the result of a distraction-based mechanism [1, 4]. Prior research has shown that engagement in a VR environment can redirect attention away from painful stimuli, thereby reducing pain perception [4, 23]. Alternatively, Holopoint at L2 and L3 may have reduced pain at the dominant bicep via distraction or a local pain inhibitory effect induced by MVPA of the arm given that ~85–95% of gameplay involved MVPA of the dominant arm [10]. Prior research has shown that exercise increases expression of endogenous opioid related substances locally at the exercising muscle, which could potentially decrease the nociceptive signal at the site of exercising muscle. Notably, arm MVPA was not associated with the magnitude of pain reduction at the bicep during gameplay; however, this could have been due to the lack of variability in arm MVPA during Holopoint (i.e., over 90% of participants at both levels spent at least 80% of gameplay in MVPA of the arm). Finally, cycling and Hot Squat, which involved the greatest cardiovascular response but less arm movement, may have elicited a central pain inhibitory effect (e.g., pain reduction in a body part distant to primary exercising muscles) induced primarily by exercise [8, 22]. Indeed, cardiorespiratory exercise of sufficient intensity activates central descending pathways involving the opioid and serotonergic systems for widespread pain inhibition [22]. Activation of these central pain inhibitory effects generally necessitates that the exercise be at least moderate intensity, with greater pain reduction at higher intensities [8, 10]. Nonetheless, the proposed mechanisms underlying hypoalgesia for each game are speculation and need to be verified with future research.

Partially supporting our first hypothesis, the results indicated that hypoalgesia at the thigh was only present for the 3 conditions eliciting the greatest cardiovascular intensity (cycling, Hot Squat, Holopoint L3). Participants completed on average 11–12 minutes of MVPA (based on HR) during Hot Squat and cycling, and 7–8 minutes of MVPA during Holopoint L3. Holopoint L2 and Relax Walk, which elicited the lowest cardiovascular intensity (~4 and 0 minutes of MVPA, respectively), did not induce hypoalgesia at the thigh although Holopoint L2 trended in the hypothesized direction. These findings suggest that hypoalgesia at the thigh may have been driven more by exercise vs. VR mechanisms. In a similar study, Evans et al. found the greatest hypoalgesic effect at the thigh following the VR game eliciting substantial whole-body movement (Hot Squat) compared to the VR games eliciting minimal whole-body movement (Beat Saber, Relax Walk) [11].

We also hypothesized that pain reduction would be related to the movement and intensity of gameplay within each active VR game. In support of this hypothesis, movement of the thigh was associated with pain reduction at the thigh for Holopoint, such that greater thigh MVPA and less thigh sedentary time were associated with greater pain reduction during this game. This finding is similar to a previous non-VR active gaming study demonstrating that increased EIH during active video gaming was associated with greater whole body MVPA and less sedentary time [24]. We did not find a relationship between the movement variables and pain reduction within Hot Squat. This lack of a relationship may have been caused by several factors. First, given the cardiovascular intensity of gameplay (important for central pain inhibitory effects) and the significant thigh movement (important for local effects) during Hot Squat, pain reduction at the thigh may have been influenced by combined local and central pain inhibitory effects during this game. Second, Hot Squat requires participants to periodically sustain a squat, which involves isometric muscular contraction of thigh muscles that would not be reflected by the accelerometer data. Thus, even though the thigh accelerometers indicated that approximately 30% of gameplay involved the thigh being sedentary, some of this sedentary time likely involved muscle contraction. These factors could have precluded a relationship emerging between any of the individual movement variables and hypoalgesia during Hot Squat.

This study had several limitations. First, while Hot Squat and cycling were matched for exercise intensity based on HR, the movements elicited by the two conditions were not identical. Future studies should compare identical physical activities with and without VR to confirm whether the separate hypoalgesic effects of VR and PA could be additive. Second, we used PPTs as the mode of experimental pain. Many different modes and tests of experimental pain exist (e.g., thermal pain vs. pressure pain; suprathreshold vs. threshold). Previous research has demonstrated that EIH can differ depending on the experimental pain test [25]. Therefore, we may have found different results with other methods of experimentally induced pain. Third, this study only included young, mostly active, healthy adults. Thus, whether our results generalize to other populations such as sedentary individuals and those with chronic pain remains unclear. Prior studies have shown that individuals with Fibromyalgia, chronic widespread pain, or chronic fatigue syndrome experience no pain reduction or even hyperalgesia following moderate to vigorous exercise [26–28]. Future research should investigate the effects of active VR games on experimental and clinical pain in these populations. Perhaps active VR games could serve as a distraction from pain symptoms while also simultaneously serving as a mode of exercise.

In conclusion, we demonstrated that non-active VR, active VR, and moderate intensity cycling reduced pressure pain sensitivity at the bicep. In contrast, only the active VR games eliciting the greatest cardiovascular response and cycling reduced pressure pain sensitivity at the thigh. Additionally, Hot Squat which had a similar exercise intensity to cycling did not induce greater hypoalgesia compared to cycling. As such, the hypoalgesic effects of VR and physical activity did not appear to be additive in this study. However, the hypoalgesic effects of the non-active VR game were not as robust or widespread compared to exercise or the active VR games, which decreased pain sensitivity at the thigh and bicep, as opposed to only the bicep. Moreover, the magnitude of pain reduction at the thigh during Holopoint appeared to be partly driven by moderate to vigorous movement of the thigh, suggesting the involvement of a local pain inhibitory mechanism driven by MVPA of the exercising muscle.

## Supporting information

S1 File Dataset. (PDF)
