# Supplementary material for: The acute hypoalgesic effects of active head-mounted display virtual reality games
Source: PLoS One. 2024 Aug 14;19(8):e0308064. doi: 10.1371/journal.pone.0308064 (PMC11324122; doi:10.1371/journal.pone.0308064)
Supplement: S1 File — (PDF) [file pone.0308064.s001.pdf]

Age	Sex (1=MALE, 2=FEMALE)	HP2_PPT_BICEP_Fam_AVG	HP2_PPT_thigh_Fam_AVG	HP2_PPT_PreVR_BICEP_AVG	HP2_PPT_PreVR_thigh_AVG	HP2_PPT_PostVR_BICEP_AVG	HP2_PPT_PostVR_thigh_AVG	HP3_PPT_BICEP_Fam_AVG
18	2	4.37	10.55	6.59	10.5	8.01	12.15	9.7
19	1	7.76	15.6	7.29	14.72	8.89	16.33	8
19	2	3.23	5.56	3.69	8.27	3.56	6.56	4.21
18	2	3.58	9.31	3.36	7.99	3.37	7.17	3.25
19	2	6.3	11.97	4.79	12.22	5.43	10.51	5.35
29	1	10.92	31.95	6.33	21.33	6.83	18.32	7.68
22	1	8.06	21.35	9.35	23.18	8.88	20.3	7.82
21	1	16.89		15.97		18.02		23.94
28	1	11.47	26.69	10.76	27.43	10.93	28.59	9.52
29	2	5.98	13.1	7.89	12.79	11.96	13.18	7.08
21	1	6.3	13.27	5.86	12.31	7.98	15.06	7.19
25	1	8.66	28.83	11.26	25.51	8.78	35.46	8.34
34	1	9.3	21.41	8.32	19.91	9.5	21.83	7.78
21	1	10.34	19.56	11.47	16.49	12.12	20.42	13.17
24	2	4.6	15.99	4.75	11.46	4.37	10.62	3.6
27	2	5.93	11.97	5.95	12.66	7.1	15.95	5.95
20	2	4.08	8.09	4.16	5.75	3.53	8.13	3.2
29	1	7.91	13.02	7.11	13.1	7.11	14.32	7.18
21	1	5.78	8.96	5.44	8.79	5.46	8.16	4.38
24	1	7.39	31.49	11.94	27.61	17.24	40.21	20.16

HP3_PPT_thigh_Fam_AVG	HP3_PPT_PreVR_BICEP_AVG	HP3_PPT_PreVR_thigh_AVG	HP3_PPT_PostVR_BICEP_AVG	HP3_PPT_PostVR_thigh_AVG	HS_PPT_BICEP_Fam_AVG	HS_PPT_thigh_Fam_AVG	HS_PPT_PreVR_BICEP_AVG	HS_PPT_PreVR_thigh_AVG
20.02	10.45	22.42	9.28	23.99	7.76	14.63	10.19	16.02
21.6	8.27	17.65	8.14	21.96	9.39	14.23	10.08	15.06
7.26	4.75	6.55	4.57	7.17	3.53	7.23	3.69	7.95
9.91	3.49	6.95	3.56	7	3.68	9.05	3.09	9.34
6.49	3.61	7.76	3.28	6.68	4.32	6.23	3.91	6.65
17.15	5.81	17.88	8.03	19.69	6.85	19.03	5.91	17.18
25.34	9.51	21.12	8.04	24.23	9.56	21.85	11.02	17.57
	23.18		22.06		12.91		20.71	
25.64	9.94	22.44	12.68	25.29	14.82	30.83	13.71	39.43
15.35	10.71	12.84	10.66	14.42	8.87	10.01	8.91	14.15
15.06	7.73	19.6	8.27	16.22	6.1	15.13	7.21	20.56
21.73	9.19	21.6	11.43	26.68	11.21	23.59	11.28	34.69
19.2	8.11	19.42	8.98	19.78	8.57	16.28	8.21	21.1
17.91	10.38	21.33	14.35	21.33	11.36	17.84	9.24	16.38
9.87	3.75	11.92	4.15	9.85	4.11	8.61	3.46	5.96
10.81	6.65	12.9	6.41	16.75	6.8	16.57	6.85	18.83
8.4	3	5.81	3.69	7.83	4.46	8.84	4.34	8.03
12.39	9.29	13.96	8.01	14.99	5.51	19.04	6.98	13.57
9.38	5.34	8.6	5.93	9.21	6.66	10.78	5.09	8.89
37.4	14.74	44.47	20.63	46.54	18.69	27.25	10.81	26.38

HS_PPT_PostVR_BICEP_AVG	HS_PPT_PostVR_thigh_AVG	RW_PPT_BICEP_Fam_AVG	RW_PPT_thigh_Fam_AVG	RW_PPT_PreVR_BICEP_AVG	RW_PPT_PreVR_thigh_AVG	RW_PPT_PostVR_BICEP_AVG	RW_PPT_PostVR_thigh_AVG	EX_PPT_BICEP_Fam_AVG
9.93	18.79	12.63	19.84	13.32	23.93	15.02	25.67	11.61
10.74	16.87	8.54	14.93	7.03	15.45	7.67	13.2	8.09
3.96	7.99	3.22	5.69	3.18	5.85	4.53	6.5	4.2
2.94	8.81	3.93	9.66	2.63	7.59	3.86	7.29	3.54
4.01	7.95	3.14	6.42	1.75	4.89	2.73	5.7	4.34
6.77	18.65	7.35	20.85	7	20.35	7.7	20.62	4.99
12.77	20.35	11.26	24.36	11.53	23.67	12.04	23.01	11.02
25.14		13.43		12.73		13.07		23.62
17.68	43.58	14.3	28.42	13.77	28.48	14.19	22.22	18.86
13.04	15.35	7.46	15.2	8.14	13.08	7.28	15.08	13.05
8.17	17.65	5.21	15.3	5.77	14.1	6.21	12.81	8.21
11.27	33.79	9.79	25.46	9.34	27.3	7.85	25.91	11.29
7.88	26.34	9.32	20.22	7.82	23.54	6.36	19.13	7.99
10.45	20.05	9.72	19.06	11.58	16.15	11.76	19.52	11.66
2.63	4.89	3.54	9.99	3.51	9.54	3.41	9.22	3.37
7.77	21.71	5.55	18.11	6.36	17.02	6.49	16.85	6.31
4.81	8.51	4.24	13.26	3.92	8.76	4.11	9.91	3.17
6.51	13.07	7.69	12.88	5.83	13.91	5.29	12	7.2
7.36	11.72	4.07	7.31	4.86	6.74	5.2	7.13	5.26
9.16	28.55	8.75	23.48	9.67	27.83	25.2	33.04	8.34

EX_PPT_thigh_Fam_AVG	EX_PPT_PreVR_BICEP_AVG	EX_PPT_PreVR_thigh_AVG	EX_PPT_PostVR_BICEP_AVG	EX_PPT_PostVR_thigh_AVG	HP2_ACTG_WRIST_PSed	HP3_ACTG_WRIST_PSed	HS_ACTG_WRIST_PSed	RW_ACTG_WRIST_PSed
27.54	15.6	28.56	15.27	35.34	2.18	1.28	37	97.57
20.47	9.1	20.6	9.44	21.65	2.56	0.9	25.86	90.91
5.34	3.74	6.02	5.31	6.12	3.33	8.96	31.75	96.67
6.55	3.3	6.78	2.28	7.57	0.77	0.38	85.4	95.39
9.04	3.05	9.2	3.58	9.94	3.97	1.54	31.67	81.31
11.89	5.07	9.94	5.93	11.69	2.05	0.26	16.13	86.04
19.81	9.67	24.11	9.49	24.57	3.59	0.77	7.04	68.25
	21.84		28.55		0.64	0.64	13.19	89.5
41.75	18.6	40.49	21.49	53.43	2.56	0.64	17.29	84.51
15.71	11.13	13.86	10.07	17.97	8.07	3.46	6.53	98.34
16.86	6.04	17.15	8.99	17.94	0.9	1.79	11.01	45.2
22.59	9.26	27.06	10.62	27.39	5.89	1.15	8.32	27.14
17.76	8.07	15.74	10.22	26.85	9.6	6.02	24.33	90.65
19.13	12.68	17.97	13.98	25.14	1.02	2.18	10.63	63.25
6.75	3.21	6.16	4.14	5.78	2.3	0.26	4.48	80.92
17.11	6.7	18.04	7.96	24.06	4.74	2.43	4.23	95.9
8.76	3.54	6.63	3.4	8.04	4.53	0.64	49.74	99.36
17.55	9.82	20.34	4.95	15.06	4.48	0.64	22.28	74.26
8.66	4.99	8.69	6.87	10.01	0.26	0.51	18.57	85.53
36.23	14.67	31.75	13.68	43.14	3.2	5.89	2.69	73.37

EX_ACTG_WRIST_PSed	HP2_ACTG_WRIST_PMVPA	HP3_ACTG_WRIST_PMVPA	HS_ACTG_WRIST_PMVPA	RW_ACTG_WRIST_PMVPA	EX_ACTG_WRIST_PMVPA	HP2_ACTG_THIGH_PSed	HP3_ACTG_THIGH_PSed	HS_ACTG_THIGH_PSed
90.14	83.61	88.22	34.06	0.26	0.77	53.14	50.26	35.98
65.43	93.09	96.41	54.16	3.07	10.76	17.16	2.53	28.94
93.98	76.18	69.27	40.2	1.54	3.46	38.41	68.5	39.69
91.04	87.96	93.59	8.96	1.79	5.25	25.26	38.54	19.97
88.48	75.8	88.48	47.82	5.76	4.87	87.07	60.82	35.9
78.23	91.29	97.31	49.94	2.43	9.73	31.11	11.4	12.93
89.12	91.68	97.06	70.93	16.01	5.63	40.46	41.49	63.76
63.89	95.77	97.31	65.94	5.51	14.72	25.1	69.57	17.67
62.36	92.57	94.24	44.05	2.43	2.05	35.72	27.78	23.69
63.25	82.07	92.06	65.04	0.51	8.45	47.5	37.13	25.61
66.97	92.7	93.98	63.64	16.13	15.62	11.78	10.76	29.32
86.3	86.56	95.77	65.3	42.38	4.61	16.13	10.37	15.49
71.19	80.28	84.51	46.73	2.69	1.54	16.77	23.18	47.12
73.5	86.94	89.24	67.61	15.49	4.74	12.8	11.65	19.97
85.92	85.02	94.88	88.48	8.19	4.35	54.67	35.98	6.91
76.06	88.73	93.21	76.82	2.69	9.73	27.66	8.83	9.73
48.7	84.88	96.54	14.08	0	29.49	68.14	32.27	50.19
75.16	88.6	97.31	50.7	10.37	9.22	36.88	7.68	28.55
69.53	93.47	96.16	54.29	8.83	7.81	38.03	26.25	54.03
46.99	92.96	88.22	66.71	9.22	35.72	13.7	29.19	42.38

RW_ACTG_THIGH_PSed	EX_ACTG_THIGH_PSed	HP2_ACTG_THIGH_PMVPA	HP3_ACTG_THIGH_PMVPA	HS_ACTG_THIGH_PMVPA	RW_ACTG_THIGH_PMVPA	EX_ACTG_THIGH_PMVPA	HP2_MVPA-HR_MIN	HP3_MVPA-HR_MIN
99.87	0	1.15	2.81	51.98	0	100	3.02	1.83
99.1	0	36.36	40.51	59.15	0	100	7.3	5.7
98.98	0	30.39	12.55	36.36	0	100	12.08	3.15
99.1	0	24.1	6.53	66.07	0	100	6.87	7.55
99.49	0	0	1.41	42.18	0	100	0	5.32
90.65	3.59	24.71	47.63	61.72	0	94.49	6.37	14.16
93.21	0	2.18	2.69	0.38	0	100	3.45	4.23
97.7	2.94	18.95	8	42.64	0	92.32		0.3
85.4	0	3.33	6.79	54.16	0.13	100	0	0
98.46	0	10.63	9.48	13.96	0	100	0.83	6.48
96.93	6.79	32.01	52.11	16.65	0	85.28	0	10
84.12	0	49.42	45.84	61.08	1.54	99.36	13.05	13.15
95.9	0	59.54	48.4	34.06	0.13	100	0.4	8.42
84.89	2.3	32.39	41.23	47.76	0.38	92.06	0	5.62
93.09	0	0.64	0.77	42.13	0	100	0	0
95.77	1.79	25.48	51.6	54.16	0	97.57	8.75	14.4
85.92	30.86	0.69	4.35	12.16	4.74	66.97	8.72	12.72
81.56	0	24.33	67.73	59.03	0.13	99.23	12.48	14.02
97.18	0.51	8.96	21.13	7.94	0	99.36	0	0
95.52	1.02	63.25	45.45	29.71	0.38	75.54	6.67	10.33

HS_MVPA-HR_MIN	RW_MVPA-HR_MIN	EX_MVPA-HR_MIN	HP2_PPT_ARM_CH	HP3_PPT_ARM_CH	HS_PPT_ARM_CH	HP2_PPT_THIGH_CH	HP3_PPT_THIGH_CH	HS_PPT_THIGH_CH
13.05	0	13.69	1.42	-1.17	-0.26	1.65	1.57	2.77
11.7	0	13.26	1.6	-0.13	0.66	1.61	4.31	1.81
	0	14.1	-0.13	-0.18	0.27	-1.71	0.62	0.04
13.5	0	9.57	0.01	0.07	-0.15	-0.82	0.05	-0.53
14.35	0	14.3	0.64	-0.33	0.1	-1.71	-1.08	1.3
13.38	0	13.82	0.5	2.22	0.86	-3.01	1.81	1.47
14.21	0	13.15	-0.47	-1.47	1.75	-2.88	3.11	2.78
13.69	0	13.97	2.05	-1.12	4.43			
4.78	0	4.11	0.17	2.74	3.97	1.16	2.85	4.15
10.73	0	10.17	4.07	-0.05	4.13	0.39	1.58	1.2
11.03	0	12.68	2.12	0.54	0.96	2.75	-3.38	-2.91
12.27	0	14.28	-2.48	2.24	-0.01	9.95	5.08	-0.9
11.02	0	10.2	1.18	0.87	-0.33	1.92	0.36	5.24
12.08	0	10.05	0.65	3.97	1.21	3.93	0	3.67
11.17	0	0	-0.38	0.4	-0.83	-0.84	-2.07	-1.07
14.28	0	14.7	1.15	-0.24	0.92	3.29	3.85	2.88
12.35	0	14.82	-0.63	0.69	0.47	2.38	2.02	0.48
10.97	0	13.98	0	-1.28	-0.47	1.22	1.03	-0.5
10.43	0	14.18	0.02	0.59	2.27	-0.63	0.61	2.83
10.5	0	2.78	5.3	5.89	-1.65	12.6	2.07	2.17

HP2= Holopoint level 2
HP3= Holopoint level 3
HS= Hot Squat
RW = Relax Walk
EX = Cycling
PPT = pressure pain threshold
FAM= familiarization trials
VR=virtual reality
AVG=average
ACTG= actigraph accelerometer
Psed= percent sedentary time
PMVPA= percent moderate to vigorous physical activity
ch= change score
